# Validation of Statistical Models for Estimating Hospitalization Associated with Influenza and Other Respiratory Viruses

**DOI:** 10.1371/journal.pone.0017882

**Published:** 2011-03-11

**Authors:** Lin Yang, Susan S. Chiu, King-Pan Chan, Kwok-Hung Chan, Wilfred Hing-Sang Wong, J. S. Malik Peiris, Chit-Ming Wong

**Affiliations:** 1 Department of Community Medicine, School of Public Health, The University of Hong Kong, Hong Kong Special Administrative Region, China; 2 Department of Paediatrics and Adolescent Medicine, The University of Hong Kong, Hong Kong Special Administrative Region, China; 3 Department of Microbiology, The University of Hong Kong, Hong Kong Special Administrative Region, China; 4 The University of Hong Kong Pasteur Research Center, Hong Kong Special Administrative Region, China; Indiana University at Bloomington, United States of America

## Abstract

**Background:**

Reliable estimates of disease burden associated with respiratory viruses are keys to deployment of preventive strategies such as vaccination and resource allocation. Such estimates are particularly needed in tropical and subtropical regions where some methods commonly used in temperate regions are not applicable. While a number of alternative approaches to assess the influenza associated disease burden have been recently reported, none of these models have been validated with virologically confirmed data. Even fewer methods have been developed for other common respiratory viruses such as respiratory syncytial virus (RSV), parainfluenza and adenovirus.

**Methods and Findings:**

We had recently conducted a prospective population-based study of virologically confirmed hospitalization for acute respiratory illnesses in persons <18 years residing in Hong Kong Island. Here we used this dataset to validate two commonly used models for estimation of influenza disease burden, namely the rate difference model and Poisson regression model, and also explored the applicability of these models to estimate the disease burden of other respiratory viruses. The Poisson regression models with different link functions all yielded estimates well correlated with the virologically confirmed influenza associated hospitalization, especially in children older than two years. The disease burden estimates for RSV, parainfluenza and adenovirus were less reliable with wide confidence intervals. The rate difference model was not applicable to RSV, parainfluenza and adenovirus and grossly underestimated the true burden of influenza associated hospitalization.

**Conclusion:**

The Poisson regression model generally produced satisfactory estimates in calculating the disease burden of respiratory viruses in a subtropical region such as Hong Kong.

## Introduction

Respiratory viruses have been associated with substantial disease burden in relation to hospitalization and mortality. Reliable estimates of such disease burden are important to determine the costs and benefits associated with prevention and control strategies. Since various respiratory pathogens cannot be distinguished from each other on clinical features and as the majority of respiratory disease is not investigated virologically, the disease burden is usually estimated by statistical models. These models have so far largely been applied to influenza and to a more limited extent, to respiratory syncytial virus (RSV). The Center of Disease Control and Prevention of the United States (USCDC) has applied a Serfling cyclical regression model to estimate the baseline mortality by incorporating the long term and seasonal trends of pneumonia and influenza (P&I) mortality [1]. This model may not be applicable to subtropical and tropical regions where seasonal trends of mortality and influenza and other respiratory virus activity are relatively unpredictable. Even in temperate regions with well isolated winter peaks of influenza and RSV, concerns have been raised about the potential confounding effect of the co-circulation of other respiratory viruses on Serfling model derived estimates of influenza disease burden [2].

Two other approaches have been used to address these concerns and also to allow disease burden estimates to be made in subtropical and tropical regions with more variable and diffuse influenza virus activity than in temperate regions. One is the rate difference model (also known as excess rate model) which defines the periods of influenza epidemics and baseline periods (based on a minimal threshold of laboratory defined virus activity) and then calculates rate differences in influenza morbidity by comparing epidemic and baseline periods [3], [4]. This model may allow some correction for confounding effects of other viruses given the well separated peaks for different respiratory viruses. Another model is the Poisson regression model [5], [6], which can adjust for confounding of seasonal trends of disease outcomes, co-circulation of other respiratory pathogens and other potential confounding factors. These two methods have been applied in temperate, sub-tropical and tropical settings with varying degrees of success, demonstrating substantial influenza associated mortality and hospitalization that is comparable among different geographic regions such as the US, Hong Kong and Singapore [3], [4], [6]–[9]. Similar approaches of rate difference and Poisson regression models have been applied to estimate the disease burden associated with RSV [10]–[13], but few has been developed for parainfluenza and adenovirus, which are less predominant causes of hospitalization than influenza and RSV.

A recent study by Thompson et al. [14] compared the performance of the Serfling cyclical regression, autoregressive integrated moving average (ARIMA) model, rate difference model and Serfling–Poisson regression model in estimating the excess mortality rates associated with influenza in the US. Both Serfling regression and ARIMA models may not be applicable to the subtropical and tropical regions as they need well-separated non-epidemic periods to define baseline levels of mortality. The latter two methods require virological surveillance data from the relevant region to be included in the analysis. The authors conclude that the Poisson regression models permit estimation of influenza associated deaths but require robust virological data while the simple rate difference models may be useful in regions with sparse viral surveillance data or complex influenza seasonality [14]. However, none of the estimates from these models have been validated using a time series of virologically confirmed cases. One recent Canadian study [15] compared a convenience sample of virologically confirmed cases of RSV and influenza in children less than two years of age to validate estimates of hospitalization rates derived from statistical methods. However, their virologically confirmed cases were collected from one single hospital which served only 10% of the population of Quebec province and it may be difficult to confidently extrapolate these data to the population denominator. Furthermore, their conclusion may not be applicable to older children or to tropical regions which have more complex influenza seasonality patterns when compared with temperate regions. There is thus a need for studies that validate such statistical estimates of disease burden estimates with directly observed and virologically confirmed outcomes. In this study we used the hospitalization data of virologically confirmed cases to validate two of the above-mentioned methods: rate difference and Poisson regression models, for estimating excess hospitalization associated with each respiratory virus.

## Methods

### Ethics Statement

This study has been approved by the Ethics Committee of Li Ka Shing Faculty of Medicine, the University of Hong Kong (EC1880-02).The consent was not required for this study as there was no personal information collected from subjects.

From October 2003 to September 2006, the study subjects were recruited from the only two public hospitals located on Hong Kong Island: Pamela Youde Nethersole Eastern Hospital (PYNEH) and Queen Mary Hospital (QMH) [16], [17]. All the pediatric patients who were admitted into these two hospitals for acute respiratory diseases (ARD) on one sampling day of each week were tested for infection of respiratory viruses by immunofluorescence and culture at the microbiology laboratory of QMH. In this study the weekly number of age-specific hospital admissions with laboratory confirmed virus infection was divided by its population denominator to obtain the “directly observed rate”. This rate was regarded as a true virus associated hospitalization rate for acute respiratory disease in our study population against which currently used models can be validated. Specifically, anonymous data on children aged <18 years with a discharge diagnosis of ARD, 460–466 or 480–487 (*International Classification of Diseases* , *9^th^ Revision*, *Clinical Modification*) (ICDCM9) from the two public hospitals were obtained from the computerized database of the Hong Kong Hospital Authority with permission. Data for each record included ICDCM9 codes for up to 4 discharge diagnoses in addition to the ARD diagnosis, age, gender, dates of admission and discharge and disposition (alive or dead).

### Rate difference model

As previously described, the period of influenza predominance was defined as a period of two or more consecutive weeks in which the weekly numbers are greater or equal to 4% of the annual number of virologically confirmed influenza A and B diagnoses and less than 2% of the annual number of RSV diagnosis [3]. For comparison, periods of at least two consecutive weeks in which both the numbers of RSV and influenza virus diagnoses were less than 2% of their annual totals were defined as periods of baseline activity for both viruses. To calculate ARD hospitalization attributable to influenza, we compared mean hospitalization rates during the periods of influenza predominance with those during the baseline periods. The estimate for the whole Hong Kong Island was made by multiplying the number of ARD hospitalizations in the two hospitals by the reciprocal of the proportion of pediatric patients served (i.e. 1/0.725). As RSV, parainfluenza and adenoviruses have less definable seasonality and their peaks usually overlapped with those of influenza (Figure S1), we could not find a satisfying definition for their baseline and predominance periods, and therefore did not apply the rate difference model to these viruses.

### Poisson Regression Model

As in our previous study, the Poisson regression model with a log link was used to model the weekly numbers of ARD admissions [6]. The Poisson model assumes that the mean of hospital admissions is equal to its variance, but this assumption was not supported in our data as the variance of hospitalization data was larger than its mean (termed as over-dispersion of variance), we therefore adopted a quasi-likelihood method, which allows greater variance than the conventional Poisson distribution, to adjust for this over-dispersion [18]. Our model differs in some respects from the Serfling-Poisson model used by Thompson and colleagues which adopted a pair of sinusoidal terms to adjust for seasonal variation of mortality [14]. To make the model more robust to the effects of less predictable seasonality, we first built a core model to control the confounders, including long-term trends, seasonal patterns of ARD admissions and meteorological factors, with natural cubic spline smoothing functions of time, weekly average temperature and relative humidity. Smoothing functions were applied to remove seasonality and long term variations which are expected to be associated with time-varying confounders. The goodness of fit of the core model or its adequacy in controlling for time-varying confounding was judged by a lack of autocorrelation in its residuals. If there was still autocorrelation after adjusting for all the potential confounders, additional auto-regressive (AR) terms of residuals were added to the core model until the updated residuals distributed randomly and independently of each other. The virus activity variables for influenza (type A and B), RSV, parainfluenza (type I, II and III) and adenovirus were then simultaneously entered into the core model. The baseline hospitalization specific for a certain virus was calculated as the expected hospitalization numbers when the weekly proportions of that virus were set equal to zero and the observed data of other variables were simultaneously entered into the Poisson model. In this baseline level that specified virus was assumed not circulating in the community while the effects on hospitalization associated with cold winter, other co-circulating respiratory viruses and other unknown seasonal factors were represented. The age-specific excess hospitalization rate for each specified virus was defined as the difference between the annual sums of observed and virus-specific baseline hospitalization for each age group divided by the age-specific population. The 95% confidence intervals of excess rates were estimated by bootstrapping the residuals of the full model 1,000 times. A detailed description for Poisson modeling approach is provided in File S1.

In our previous study, the virus activity was measured by the weekly proportions of specimens positive for each virus (proportion variable), respectively. We calculated the proportions based on the virology data from the microbiology laboratory of QMH which covered all the age groups on Hong Kong Island. We also repeated the analysis with the weekly numbers of positive specimens as an alternative proxy for virus activity (number variable) in our Poisson models.

The log-link Poisson models have been previously criticized for assuming that the numbers of hospital admissions increase exponentially with the proportions of positive isolates [19]. We therefore also built a Poisson model with an identity link, in which the hospital admissions increase proportionally with unit increase of virus activity. The age-specific excess hospitalization rate associated with influenza derived from each of these Poisson models was compared with the directly observed rate of influenza and the model that provided the most accurate estimate for influenza was chosen for analysis of other respiratory viruses.

## Results

The virologically confirmed, population-based age-specific hospitalization rates for each respiratory virus during these three study years are shown in Table 1. Among these viruses, influenza is the only one with frequent antigenic changes. Antigenic drift variants that appeared during the study period were A/California/7/04-like (H3N2) virus in 2004–05 and A/HK/2652/06-like (H1N1) virus in 2005–06 [16]. However, the H1N1 A/HK/2652/06-like virus did not start to circulate until the second half of 2006, and therefore had minimal impact on this study which ended in September 2006.

**Table 1:** Comparison of influenza associated ARD hospitalization rate (per 10,000) estimated from the rate difference model, log-link Poisson and identity-link Poisson regression models with virologically confirmed hospitalization (Directly observed) in patients <18 years on Hong Kong Island.

Age group	Directly observed	Rate difference	Log-link Poisson (proportion)^a^	Log-link Poisson (case number)^b^	Identity-link Poisson (proportion)^a^	Identity-link Poisson (case number)^b^
**2003–2004**						
<1	77.8	11.2	128.7	72.0	104.7	79.6
		(−9.2, 31.5)	(41.5, 207.3)	(22.9, 120.0)	(−15.3, 223.6)	(8.7, 150.5)
1-<2	95.5	2.2	145.9	89.6	178.5	121.4
		(−3.5, 17.5)	(64.4, 217.7)	(46.7, 136.3)	(51.8, 305.1)	(51.1, 192.5)
2-<5	67.7	17.0	81.6	47.9	143.1	88.4
		(8.7, 25.1)	(47.9, 111.8)	(27.8, 66.9)	(86.9, 199.3)	(53.7, 123.1)
5-<10	18.2	2.6	18.9	7.5	22.5	17.0
		(−0.2, 5.5)	(8.7, 28.3)	(1.9, 13.6)	(1.4, 43.5)	(4.4, 29.4)
10-<18	2.7	1.3	5.2	2.3	6.7	4.8
		(0.3, 2.2)	(1.4, 8.5)	(0.1, 4.5)	(0.9, 12.5)	(1.2, 8.4)
**2004–2005**						
<1	103.8	NA	125.3	109.2	81.9	94.3
			(41.0, 203.5)	(34.7, 177.5)	(−11.2, 175.0)	(9.9, 178.7)
1-<2	68.2	NA	145.3	137.6	139.3	145.3
			(62.9, 220.9)	(70.5, 208.2)	(40.8, 238.8)	(61.2, 229.4)
2-<5	84.6	NA	82.6	72.5	111.7	102.8
			(48.1, 114.1)	(42.2, 100.4)	(67.7, 155.1)	(62.4, 143.2)
5-<10	28.2	NA	26.4	16.7	17.6	19.9
			(12.0, 39.6)	(3.9, 30.1)	(1.1, 33.9)	(5.2, 34.8)
10-<18	4.6	NA	5.4	3.8	5.1	5.4
			(1.4, 9.0)	(0.2, 7.3)	(0.7, 9.5)	(1.4, 9.5)
**2005–2006**						
<1	38.9	9.2	86.1	64.0	61.5	64.0
		(−51.2, 69.6)	(25.8, 141.4)	(19.7, 107.0)	(−8.6, 131.6)	(7.4, 121.8)
1-<2	54.6	58.4	116.1	95.4	107.1	100.8
		(15.1, 101.4)	(48.6, 180.1)	(48.6, 145.9)	(31.5, 182.8)	(42.3, 159.4)
2-<5	63.5	0.8	67.0	51.9	85.4	72.9
		(−36.2, 37.6)	(38.1, 94.0)	(30.2, 73.6)	(51.9, 118.9)	(44.0, 101.2)
5-<10	24.6	3.8	21.6	11.4	13.3	13.9
		(−2.5, 10)	(9.6, 33.1)	(2.9, 20.6)	(0.8, 25.9)	(3.7, 24.3)
10-<18	8.2	0.1	5.2	3.1	3.9	3.9
		(−1.6, 1.9)	(1.3, 8.9)	(0.2, 6.0)	(0.5, 7.2)	(1.0, 6.7)

The 95% confidence interval for each estimate is shown in bracket.

Note: ^a^ Models with the proportions of positive specimens as proxy for influenza activity.

b Models with the numbers of positive specimens as proxy.

### Influenza associated ARD hospitalization rates

#### Rate difference model

**Figure 1:**
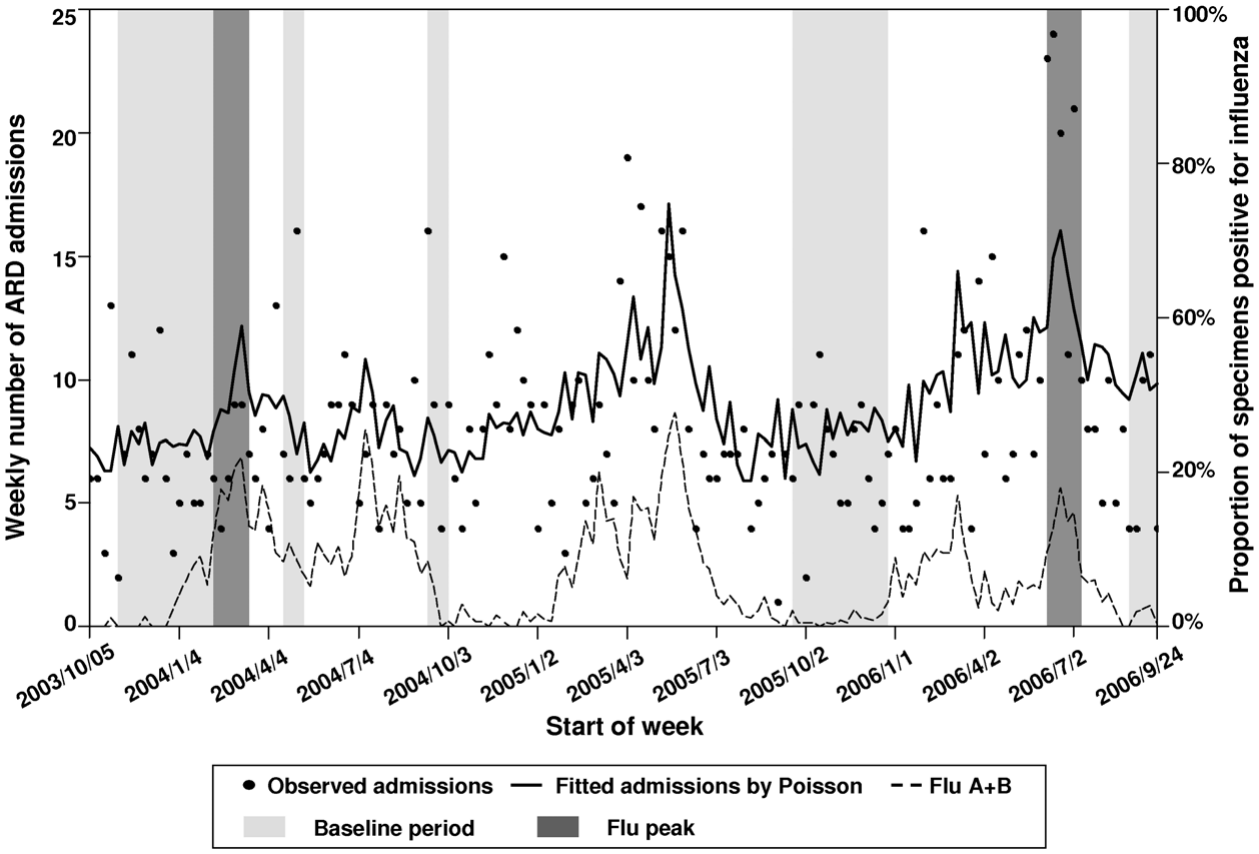
Observed (dots) and predicted numbers (solid line) of ARD hospitalization in the 1<-2 age group. The weekly proportions of specimens positive for influenza are shown in broken line. The baseline and predominance periods defined by the Rate Difference model are highlighted in light grey and dark grey colors.

**Figure 2:**
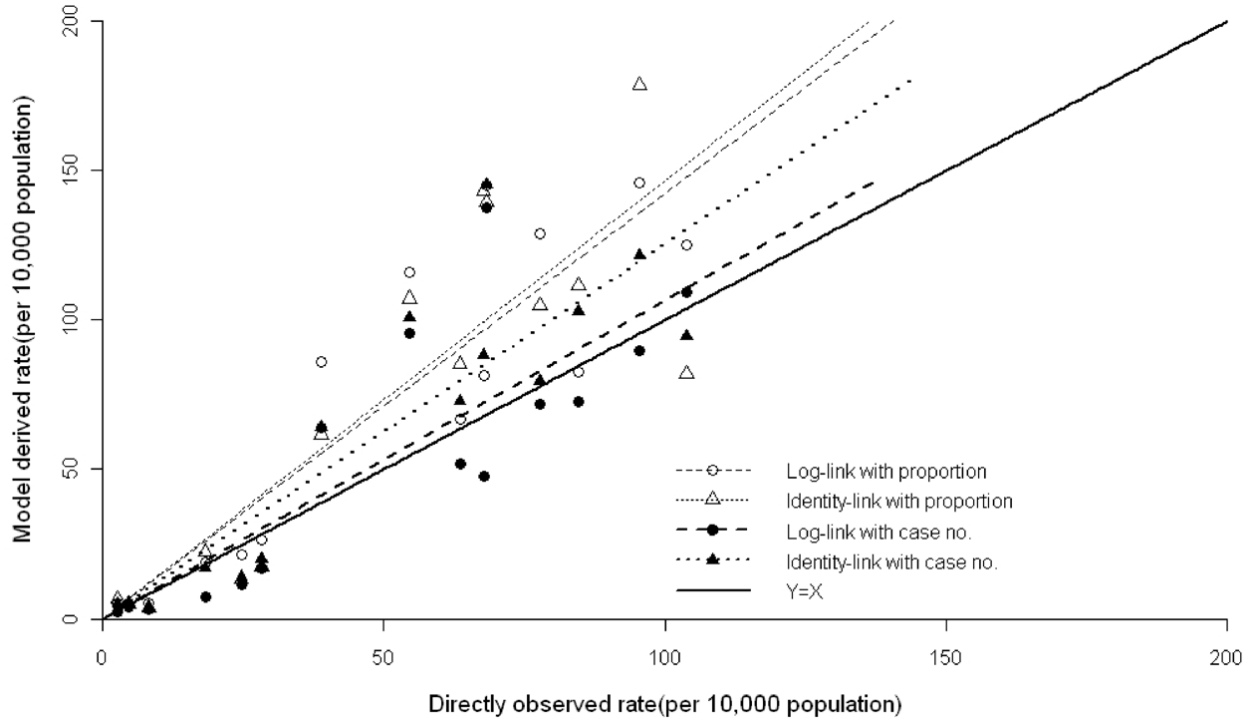
Comparison of age-year specific influenza-associated excess ARD hospitalization rates. The estimates from the log-link Poisson regression with the proportion variables (open circle) and identity-link Poisson with the proportion variables (open triangle) and log-link Poisson with the case number variables (solid circle) and identity-link Poisson models with the case number variables (solid triangle) were plotted against directly observed hospitalization rates with diagnosis of influenza infection. A linear regression line was separately fitted to the estimates derived from each model. The thick black line is the diagonal for y = x.

On the basis of the study definition, there were four weeks in which influenza predominated in 2003–04 and 2004–05 seasons whereas there were none in 2004–05 (Figure 1). In 2003–04, 2004–05 and 2005–06, there were 17, 6 and 16 weeks respectively, in which neither influenza virus nor RSV was active (baseline period). The rate difference model could not be used for the 2004–05 season since an influenza predominance period could not be defined. When compared to the directly observed virologically confirmed influenza hospitalization rates for each age group, the rate difference model failed to produce estimates that closely matched the directly observed incidence rates for the other two years (Table 1 and Figure 2).

#### Poisson Regression model

The log-link Poisson models with the proportion variables yielded the estimates close to the directly observed rates for the children older than two years, but the estimates tended to much higher for the <1 and 1 to <2 age groups (Table 1). The identity-link function overestimated the true disease burden to a greater extent than did the log-link function in most age-year categories, no matter what influenza proxy variable was used. The influenza effect for each age group was found to be statistically significant (*p*<0.05) in all the models, the only exception being the <1 age group in the identity-link Poisson models with proportion variables (Table 1). Overall, the log-link models with the number variables provided the estimates closest to the directly observed rates, despite underestimation observed for the 2–5 and 5–10 age groups (Figure 2). The log-link Poisson models with the number variables were chosen for further analysis for other respiratory viruses.

### ARD hospitalization rates associated with other respiratory viruses

In the age group younger than one year, the estimates of excess hospitalization rates for parainfluenza were close to those directly observed from young children, but those for RSV tended to be lower for the 2003–2004 and 2005–2006 seasons (Table 2). For children older than 1 year of age, the estimates for excess hospitalization associated with RSV or parainfluenza derived from the log-link Poisson model with the number variables had wide confidence intervals and were markedly deviant from the directly observed rates. The estimates for adenovirus associated hospitalization were unreliable at all ages.

**Table 2:** The ARD hospitalization rate (per 10,000) associated with respiratory syncytial virus (RSV), parainfluenza and adenovirus in patients <18 years on Hong Kong Island.

Age	2003–04		2004–05		2005–06	
	Directly observed	Poisson regression(95% CI)	Directly observed	Poisson regression(95% CI)	Directly observed	Poisson regression(95% CI)
**RSV**						
<1	155.6	135.3	168.5	234.5	233.4	157.4
		(77.5, 192.0)		(136.5, 328.9)		(89.8, 222.6)
1-<2	81.8	37.8	163.7	72.2	54.6	50.4
		(−23.7, 10 4.4)		(−49.3, 191.2)		(−32.4, 142.2)
2-<5	59.2	12.2	38.1	24.4	33.9	17.1
		(−14.2, 37.6)		(−28.5, 71.9)		(−19.7, 52.6)
5-<10	1.8	9.2	3.6	23.8	1.8	17.8
		(0.6, 16.3)		(1.6, 41.4)		(1.0, 32.3)
10-<18	0.0	2.7	0.0	5.4	0.0	4.8
		(−0.2, 5.2)		(−0.4, 10.0)		(−0.3, 9.1)
**Parainfluenza**						
<1	64.8	70.9	103.7	121.6	90.8	107.0
		(7.6, 128.7)		(37.2, 196.1)		(1.2, 195.5)
1-<2	81.8	−16.3	150.1	4.2	54.6	−25.2
		(−92.6, 54.8)		(−95.2, 95.2)		(−150.4, 88.2)
2-<5	29.6	17.6	50.8	13.7	63.5	48.0
		(−9.3, 43.5)		(−33.9, 57.0)		(−2.0, 97.2)
5-<10	5.5	−4.6	3.6	−6.8	9.1	−7.6
		(−13.9, 4.3)		(−24.6, 10.4)		(−30.2, 15.3)
10-<18	0.9	−2.2	0.0	−2.7	0.0	−4.1
		(−5.6, 1.0)		(−7.3, 1.8)		(−11.9, 3.1)
**Adenovirus**						
<1	13.0	−3.3	77.8	−16.1	25.9	−7.4
		(−30.5, 25.1)		(−146.4, 112.9)		(−68.9, 55.3)
1-<2	13.6	14.8	54.6	69.7	13.6	35.1
		(−14.8, 44.4)		(−73.1, 194.6)		(−34.2, 103.5)
2-<5	38.1	−3.4	59.2	−16.6	38.1	−10.5
		(−17.1, 8.8)		(−85.6, 42.8)		(−53.2, 27.6)
5-<10	1.8	1.2	21.8	8.1	9.1	4.7
		(−2.8, 4.9)		(−19.2, 29.6)		(−10.6, 17.6)
10-<18	0.0	0.5	0.0	2.3	0.9	1.7
		(−0.8, 1.7)		(−4.0, 7.7)		(−2.7, 5.9)

The estimates from the log-link Poisson models were compared with virologically confirmed hospitalization (Directly observed).

## Discussion

As previously suspected, the rate difference model is not applicable when influenza does not appear as a sharp peak with reasonable separation from RSV [3]. We further demonstrate that this model greatly underestimates the actual disease burden (even when influenza predominant periods are separated from periods of RSV circulation) if significant influenza activity exists outside the predominant peak of influenza virus activity, as commonly occurs in subtropics and tropics. In addition to RSV, other common respiratory viruses can also have significant confounding effects, especially on hospitalization of children, and these cannot be readily accounted for in this model.

In Poisson regression models, the baseline levels of influenza are predicted from a model fitted to observed numbers of hospital admissions. The excess hospitalization estimate is robust to potential confounding effects due to uncontrolled individual factors that do not change over a short period of time, such as smoking status and preexisting chronic conditions [20]. Given the relatively broad and variable seasonality of influenza in the tropical and subtropical regions such as Hong Kong, it is not surprising that the Poisson regression model which uses a non-parametric smoothing function for modeling any pattern of weekly hospitalization outperformed the rate difference model in our study. Additionally, the Poisson regression model allows estimation of disease burden while more efficiently adjusting for co-morbidity caused by other respiratory viruses and also for confounding of seasonal variations of hospitalization and meteorological conditions [6]. However, the Poisson regression model has been criticized for being potentially inadequate in adjustment for confounding factors, which would result in allocating more variation of hospital admissions to explanatory variables (influenza virus activity in our model) than they deserve and thereby overestimate the influenza effects [21]. In this study, we carefully checked the adequacy of core models in terms of adjusting for confounding factors. If autocorrelation of weekly hospitalization data was still detected in the residuals after adjusting for confounding factors by smoothing, this would suggest that some unobservable confounding might remained unadjusted for. We then would further remove the autocorrelation by adding AR terms of the residuals of core models. In this way, the long-term and seasonality associated confounding factors were expected to be well controlled. The close match between Poisson regression estimates and directly observed numbers suggested that our control strategy for confounding is appropriate and sufficient.

The debate over log-link Poisson models has focused on whether it is appropriate to assume the multiplicative risk for hospitalization or mortality associated with the unit increase of proportions of positive specimens [9]. Here we did a sensitivity analysis by using the identity-link Poisson regression model which assumes the additive risks associated with influenza virus activity. The results showed that the log-link Poisson generally returned estimates smaller than those from the identity-link models. Thompson et al. also found that these two link functions produced the similar results [9]. But the lack of a clear biological rationale has made it difficult to choose the proper link function and there are no epidemiological studies that could provide solid evidence to support a linear or log-linear relationship between influenza cases and hospital admissions. As the log-link function ensures a non-negative estimate for predicted hospital admission numbers, the log-link Poisson regression model is probably more appropriate for influenza disease burden studies.

In this study we adopted the quasi-likelihood method in Poisson models to control over-dispersion of variance in hospitalization data. The negative binomial model, which is a generalized form of Poisson models and addresses the over-dispersion problem by a scale parameter has also been introduced into the influenza disease burden study [8], [15]. We then built the negative binomial model to estimate influenza associated hospitalization rates of each age-disease category. The results showed that the estimates of negative binomial models with a log-link or identity-link tended to be slightly larger than those of log-link Poisson models in the age groups younger than 5 years, but the log-link negative binomial models markedly underestimated the rates in the 5–10 age group, and influenza effects were found not statistically significant in the 0–1 and 5–10 age groups in the identity-link negative binomial models (data not shown). These findings suggest the quasi-likelihood methods performed better than the negative binomial regression in terms of producing estimates closer to the directly observed data.

The optimal indicator of virus activity in such studies has also been contentious. We have previously used the proportion of specimens positive for influenza as proxy for influenza virus activity. In this study, we carried out sensitivity analysis by replacing the positive proportions with the numbers of positive specimens as the influenza virus activity variable into the models. Both the log-link and identity-link models with the number variable generally yielded estimates closer to the directly observed rates, compared with the models with the proportion variable. Our virology data were obtained from one laboratory which consistently received 100 to 200 specimens for respiratory virus diagnosis each week from the patients admitted with acute respiratory disease and these specimen numbers were driven by clinical need and were not capped in any way. However, data from a community influenza-like-illness surveillance program that covers a city or even larger area, the influenza case numbers may be subject to bias introduced by pre-determined targets of numbers to be collected each week. Alternatively, changes in the numbers of sentinel sites or in health seeking behavior may trigger artificial changes in specimen numbers. Therefore, the proportion of positive specimens is likely to be a more robust indicator in such situations.

A limitation of this study is that the nasopharyngeal specimens were tested by immunofluorescence and viral culture, not by the more sensitive polymerase chain reaction (PCR). We found that immunofluorescence and culture under-estimates influenza cases by approximately 10% and RSV by 13% [23]. Other studies reported a 10% to 20% increase of samples positive for RSV and parainfluenza when PCR was used [24], [25]. Hence the directly observed rates in the present study may underestimate the true disease burden of these respiratory viruses. Even if we take into account this potential underestimation of directly observed rates by multiplying a factor of 10%, the log-link Poisson model with the number of specimen variable remained as the best model that offered the estimates closest to the directly observed rates of influenza and RSV, and the excess rates for parainfluenza in the <1 age group were almost equal to the true disease burden.

Although parainfluenza and adenovirus caused substantial hospitalizations of children in our study, the Poisson model did not return reliable estimates for these two respiratory viruses, with the exception of parainfluenza infection in those <1 year of age. The poor estimates in older children could be due to relatively low proportions of samples positive for parainfluenza and adenovirus from the surveillance network (Figure S1). Compared to influenza and RSV, these two viruses exhibit less clearly defined seasonal variations, which may further increase the difficulty in relating the variation of hospitalizations to the weekly proportions of parainfluenza and adenovirus.

In our study, the rate difference model tends to provide smaller estimates of influenza associated disease burden than does the Poisson model. Interestingly, Thompson et al. found that the rate difference model consistently returned higher estimates than the Poisson model in the US [14]. This could be a result of different definitions adopted by the two studies. Their study did not consider co-circulation of RSV when they defined baseline and epidemic periods, largely owing to the overlapping seasonal peaks of these two viruses in the US [22]. Moreover, the well defined single peak of influenza in winter in the US would allow a more accurate estimate from the rate difference model that in a tropical or subtropical region where the virus activity is more dispersed throughout the year.

Contrary to our findings, the Canadian study by Gilca et al. found that estimates derived from both log-link Poisson and rate difference models significantly underestimated the pneumonia and influenza admissions with laboratory confirmed influenza in children of two years old or younger [15]. Their study has the limitation that laboratory-confirmed diagnoses of influenza and RSV came from a convenience sample from one hospital that served 10% of Quebec children and may not represent the pediatric RSV/influenza epidemiology of the province. In contrast, our study is based on the data directly derived from 72.5% of all hospitalizations in Hong Kong Island, i.e. the population denominator. Gilca et al. adjusted for interaction between influenza and RSV by adding a product term into their Poisson models. We did a sensitivity analysis with the same approach and the results showed that influenza effects changed only marginally and the RSV estimates remained nearly unchanged (data not shown). Therefore, we did not need to include any interaction term in our full models.

Poisson regression models very accurately estimated the excess rate for influenza in the 2–18 year old age groups, but the influenza estimates for the children younger than two years were much higher than the directly observed rates. Interestingly, the estimates for RSV were accordingly lower in the young children. As the peaks of influenza and RSV occasionally overlapped in the study period, it is possible that such under- and over-estimate could be the result of multicollinearity between virus variables in our Poisson models. We then did a sensitivity analysis by assessing the effects of single virus without adjustment for co-circulation of other viruses, i.e. only one virus variable being entered into the model. The results showed that the influenza estimates slightly increased after model change with the exception of the 2-<5 age group. The RSV associated rates slightly increased for the 1-<2 and 5<-10 age groups and but decreased for the children younger than 1 year old (Table S1). The Poisson estimates for parainfluenza and adenovirus without adjustment for co-circulation were dramatically decreased (compared to the full models) but this is more likely due to the unstable nature of these estimates as shown in their wide confidence intervals. The results indicate a mild extent of collinearity between influenza and RSV variables, which may not significantly affect our estimates. Some of the confidence intervals for estimated age-specific rates of influenza associated hospitalization tended to be rather wide, presumably due to the fact that the study covered a period of only three years. We would expect to get narrower confidence intervals with longer time series, although it is difficult to sustain the systematic virological diagnosis of all ARD admissions on an ongoing basis to provide the virologically confirmed independent validation of the estimates.

In conclusion, Poisson regression modeling is applicable to the assessment of disease burden due to influenza associated hospitalization in children. It also yields reasonable estimates for RSV and parainfluenza in children <1 year of age, but performs less well for the older children. None of these methods provided reliable estimates of disease burden for adenovirus hospitalization. Although mortality was not assessed and could not be validated in this study, we could infer that the good correlation between the output of the Poisson model and virologically confirmed hospitalization will also be applicable to mortality estimates. Such an approach to estimation of influenza disease burden requires long-term virological surveillance data and thereby restricts its application in some geographic regions. However, with the heightened attention on influenza arising from the threat of avian influenza H5N1, and now from the recent pandemic H1N1, such virological data is becoming increasingly available in many countries making Poisson regression modelling feasible in an increasing number of contexts.

## Supporting Information

Figure S1 **Proportions of specimens positive for common respiratory viruses.** Data were collected from the influenza surveillance network of Hong Kong Island in the study period.
(TIF)

Table S1 **Excess hospitalization rates associated with respiratory viruses estimated from the Poisson models without adjusting for co-circulation of other respiratory viruses.**
(DOC)

File S1 **Multiplicative model.**
(DOC)
