# Supplementary figures and images for: Validation of Statistical Models for Estimating Hospitalization Associated with Influenza and Other Respiratory Viruses
Source: PLoS One. 2011 Mar 11;6(3):e17882. doi: 10.1371/journal.pone.0017882 (PMC3055891; doi:10.1371/journal.pone.0017882)

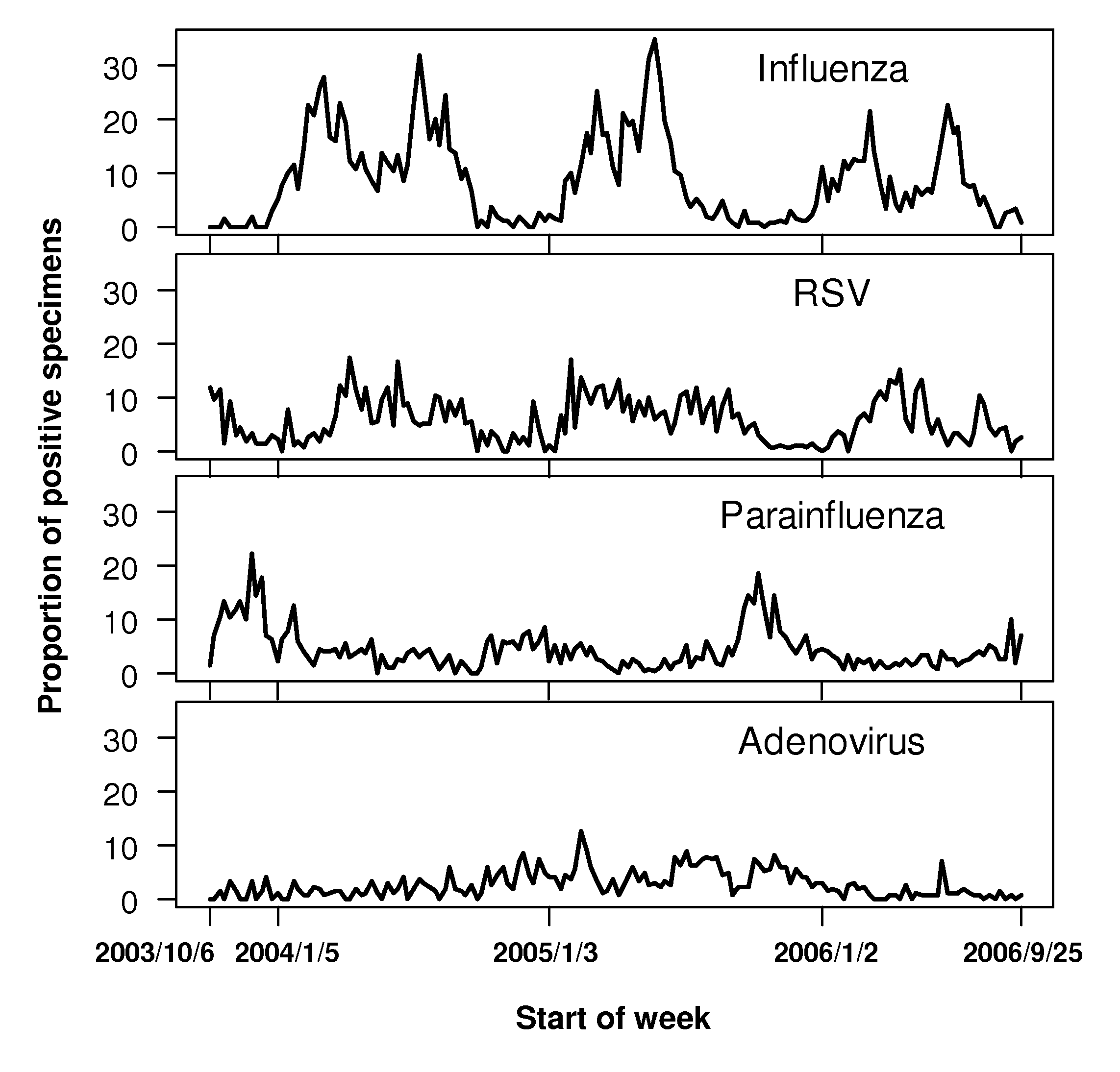

Supplement: Figure S1 — Proportions of specimens positive for common respiratory viruses. Data were collected from the influenza surveillance network of Hong Kong Island in the study period. (TIF) [file pone.0017882.s001.tif]
