# Supplementary material for: Validation of Statistical Models for Estimating Hospitalization Associated with Influenza and Other Respiratory Viruses
Source: PLoS One. 2011 Mar 11;6(3):e17882. doi: 10.1371/journal.pone.0017882 (PMC3055891; doi:10.1371/journal.pone.0017882)
Supplement: Table S1 — Excess hospitalization rates associated with respiratory viruses estimated from the Poisson models without adjusting for co-circulation of other respiratory viruses. (DOC) [file pone.0017882.s002.doc]

Table S1. Excess hospitalization rates associated with respiratory viruses estimated from the Poisson models without adjusting for co-circulation of other respiratory viruses.

| **Age** | **2003-04** | | **2004-05** | | **2005-06** | |
| --- | --- | --- | --- | --- | --- | --- |
| **Influenza** | |  |  |  |  |  |
| < 1 | 82.9 | (32.7, 130.9) | 125.3 | (49.6, 197.3) | 75.0 | (29.5, 119.3) |
| 1-<2 | 96.3 | (56.3, 139.2) | 147.8 | (86.7, 215.8) | 102.6 | (60.3, 149.5) |
| 2-<5 | 43.5 | (23.4, 62.0) | 65.4 | (35.7, 94.5) | 48.0 | (26.3, 71.0) |
| 5-<10 | 9.2 | (3.1, 15.1) | 19.4 | (6.5, 32.6) | 13.9 | (4.7, 23.5) |
| 10-<18 | 3.1 | (1.0, 5.2) | 4.8 | (1.5, 8.2) | 4.1 | (1.3, 6.9) |
| **RSV** | |  |  |  |  |  |
| < 1 | 122.2 | (67.6, 181.1) | 214.7 | (120.4, 314.0) | 145.1 | (79.9, 217.7) |
| 1-<2 | 65.9 | (5.9, 126.6) | 119.8 | (11.0, 225.2) | 89.1 | (8.1, 174.7) |
| 2-<5 | 17.6 | (-9.3, 43.0) | 33.3 | (-18.4, 80.2) | 25.0 | (-13.1, 60.5) |
| 5-<10 | 11.0 | (3.2, 18.1) | 27.6 | (8.2, 44.8) | 21.6 | (6.3, 35.5) |
| 10-<18 | 3.6 | (1.1, 5.8) | 6.9 | (2.1, 10.8) | 6.4 | (1.9, 10.6) |
| **Parainfluenza** | |  |  |  |  |  |
| < 1 | 15.3 | (-45.8, 72.0) | 45.9 | (-47.2, 126.6) | 19.7 | (-81.2, 115.6) |
| 1-<2 | -40.0 | (-111.8, 28.1) | -42.5 | (-143.6, 56.9) | -71.1 | (-189.1, 44.1) |
| 2-<5 | 2.9 | (-24.4, 27.4) | -0.6 | (-50.5, 41.6) | 15.8 | (-36.8, 62.4) |
| 5-<10 | -6.9 | (-15.7, 1.6) | -12.2 | (-30.5, 5.7) | -13.3 | (-34.9, 8.4) |
| 10-<18 | -2.9 | (-6.0, 0.1) | -4.3 | (-9.1, 0.7) | -6.1 | (-13.3, 1.2) |
| **Adenovirus** | |  |  |  |  |  |
| < 1 | -5.5 | (-36.0, 21.8) | -27.3 | (-172.5, 96.8) | -13.5 | (-81.2, 49.2) |
| 1-<2 | 8.9 | (-23.0, 36.3) | 39.9 | (-111.3, 163.1) | 21.6 | (-57.6, 90.9) |
| 2-<5 | 0.5 | (-12.2, 12.2) | 1.8 | (-63.0, 57.0) | 1.3 | (-40.1, 36.8) |
| 5-<10 | 0.0 | (-3.5, 3.6) | -0.4 | (-24.2, 23.3) | -0.2 | (-13.7, 13.9) |
| 10-<18 | 0.1 | (-1.1, 1.3) | 0.7 | (-5.6, 6.2) | 0.5 | (-4.2, 4.9) |
