# Supplementary material for: Validation of Statistical Models for Estimating Hospitalization Associated with Influenza and Other Respiratory Viruses
Source: PLoS One. 2011 Mar 11;6(3):e17882. doi: 10.1371/journal.pone.0017882 (PMC3055891; doi:10.1371/journal.pone.0017882)
Supplement: File S1 — Multiplicative model. (DOC) [file pone.0017882.s003.doc]

**File S1**

Multiplicative model

We first built a core model to adjust for confounding factors including seasonal trends of hospital admissions, temperature and relative humidity. The core model has the following typical form:

$$\log(E(y_{t}))=ns(t,df)+ns(temperature_{t},df)+ns(humidity_{t},df)$$

Here *E(y_t_)* denotes the expected hospital admission numbers at week t. *ns* represents the natural spline smoothing function for confounders. The degrees of freedom (*df*) for smoothing functions were chosen as the combination that gave the smallest generalized cross-validation (GCV) scores. In this study, *df* was set as 2 for time, 2 for temperature and 2 for relative humidity for all the models, with the only exception of the 2-5 age group which had all the *df* set at 3. The partial autocorrelation function (PACF) plots of residuals from each core model were then examined for any correlation between the residuals at any given week and those up to 4 weeks before (autocorrelation). If autocorrelation did exist, we would add up to 3 autoregressive terms to the core model [1].

The variables for virus activity (weekly proportions of positive specimens or numbers of laboratory confirmed cases) were subsequently added into the core model to get the final model as follows:

$$\begin{matrix} \log(E(y_{t}))=\beta_{0}+\beta_{1}flu_{t}+\beta_{2}RSV_{t}+\beta_{3}adenovirus_{t}+\beta_{4}paraflu_{t} \\ +ns(t,df)+ns(temperature_{t},df)+ns(humidity_{t},df) \end{matrix}$$

The excess hospitalization associated with influenza was defined as the difference between the observed hospitalization and the predicted hospitalization in the absence of virus activity (the variables of other viruses remain as observed data). The excess rate was then calculated as excess hospitalization divided by the total population of each age group on the Hong Kong Island. We adopted a bootstrapping method to calculate the 95% confidence interval (CI) for excess rate [2]. Specifically, we bootstrapped the residuals from the final model and added them back to the predicted hospitalization data with observed covariates to generate a new dataset which would be fitted with the same model again to obtain a new estimate for excess rate. This procedure was repeated for 1000 times, and then 2.5 and 97.5 percentiles of estimates constructed the 95% CI for estimated excess rate.

Additive models

Similar procedure as developing the above multiplicative models was taken to build the core models and final additive models as follows:

$$\begin{matrix} E(y_{t})=\beta_{0}+\beta_{1}flu_{t}+\beta_{2}RSV_{t}+\beta_{3}adenovirus_{t}+\beta_{4}paraflu_{t} \\ +ns(t,df)+ns(temperature_{t},df)+ns(humidity_{t},df) \end{matrix}$$

We built the multiplicative (log-link) and additive models (identity-link) by assuming the hospitalization data follow the Poisson or negative binomial distribution. The overdispersion of hospitalization data (the variance of data is larger than the mean) was adjusted by a quasi-likelihood method in Poisson models [3]. In negative binomial distribution, this overdispersion was taken into account by a scale parameter [4].

References

1. Brumback BA, Ryan LM, Schwartz JD, Neas LM, Stark PC et al. (2000) Transitional regression models, with application to environmental time series. J Am Stat Assoc 95:16-27.

2. Davison AC, Hinkley DV. (1997) Bootstrap Methods and Their Application. Cambridge University Press.

3. Carter RE, Lipsitz SR, Tilley BC. (2005) Quasi-likelihood estimation for relative risk regression models. Biostat 6:39-44.

4. Hastie TJ, Tibshirani RJ. (1990) Generalized additive models. London: Chapman and Hall.
